# Assessment of brain beta-amyloid deposition in transgenic mouse models of Alzheimer’s disease with PET imaging agents ^18^F-flutemetamol and ^18^F-florbetaben

**DOI:** 10.1186/s12868-018-0447-7

**Published:** 2018-07-27

**Authors:** Hye Joo Son, Young Jin Jeong, Hyun Jin Yoon, Sang Yoon Lee, Go-Eun Choi, Ji-Ae Park, Min Hwan Kim, Kyo Chul Lee, Yong Jin Lee, Mun Ki Kim, Kook Cho, Do-Young Kang

**Affiliations:** 1 Department of Nuclear Medicine, Dong-A University Medical Center, Dong-A University College of Medicine, 26 Daesingongwon-ro, Seo-gu, Busan, 602-812 Korea; 2 Institute of Convergence Bio-Health, Dong-A University, Busan, Korea; 3 Division of RI-Convergence Research, Korea Institute of Radiological and Medical Sciences, Seoul, Korea; 4 Pohang Center of Evolution of Biomaterials, Pohang Technopark, Pohang, Korea

**Keywords:** PET/CT imaging, Alzheimer’s disease, transgenic mouse model, ^18^F-flutemetamol, ^18^F-florbetaben

## Abstract

**Background:**

Although amyloid beta (Aβ) imaging is widely used for diagnosing and monitoring Alzheimer’s disease in clinical fields, paralleling comparison between ^18^F-flutemetamol and ^18^F-florbetaben was rarely attempted in AD mouse model. We performed a comparison of Aβ PET images between ^18^F-flutemetamol and ^18^F-florbetaben in a recently developed APPswe mouse model, C57BL/6-Tg (NSE-hAPPsw) Korl.

**Results:**

After an injection (0.23 mCi) of ^18^F-flutemetamol and ^18^F-florbetaben at a time interval of 2–3 days, we compared group difference of SUVR and kinetic parameters between the AD (n = 7) and control (n = 7) mice, as well as between ^18^F-flutemetamol and ^18^F-florbetaben image. In addition, bio-distribution and histopathology were conducted. With visual image and VOI-based SUVR analysis, the AD group presented more prominent uptake than did the control group in both the ^18^F-florbetaben and ^18^F-flutemetamol images. With kinetic analysis, the ^18^F-florbetaben images showed differences in K1 and k4 between the AD and control groups, although ^18^F-flutemetamol images did not show significant difference. ^18^F-florbetaben images showed more prominent cortical uptake and matched well to the thioflavin S staining images than did the ^18^F-flutemetamol image. In contrast, ^18^F-flutemetamol images presented higher K1, k4, K1/k2 values than those of ^18^F-florbetaben images. Also, ^18^F-flutemetamol images presented prominent uptake in the bowel and bladder, consistent with higher bio-distribution in kidney, lung, blood and heart.

**Conclusions:**

Compared with ^18^F-flutemetamol images, ^18^F-florbetaben images showed prominent visual uptake intensity, SUVR, and higher correlations with the pathology. In contrast, ^18^F-flutemetamol was more actively metabolized than was ^18^F-florbetaben (Son et al. in J Nucl Med 58(Suppl 1):S278, 2017].

**Electronic supplementary material:**

The online version of this article (10.1186/s12868-018-0447-7) contains supplementary material, which is available to authorized users.

## Background

Recently, Aβ imaging with ^18^F labeled radiotracers has been widely used for patients with Alzheimer’s disease (AD). ^18^F-flutemetamol is ^18^F labeled analogue of ^11^C-PiB produced by GE Healthcare (Buckinghamshire, UK) [2]. It has been useful in differentiating between patients with AD and healthy subjects with high specificity (96%) and sensitivity (93%) in the detection of AD, as well as high test–retest reliability [3, 4]. ^18^F-florbetaben is an ^18^F labeled polyethylene glycol stilbene derivative showing high in vitro affinity and specificity for β-amyloid plaques [3].

^18^F-florbetaben and ^18^F-flutemetamol are widely used for the diagnosis and monitoring of AD in a routine medical field. However, there are many unknown issues regarding the difference in tracer dynamics and biodistribution between ^18^F-flutemetamol and ^18^F-florbetaben. Because of the difficulty conducting the direct comparison between two tracers for humans due to the weighted exposure to radiation, a preclinical animal study is a good alternative option for a baseline study.

Recently, several imaging studies using newly developed ^18^F labeled Aβ PET tracers were reported in AD mouse models. In a previous study, the in vivo ^18^F-flutemetamol binding of Aβ deposits was tested in various AD mouse models [5]. In old APP23 mice, significant ^18^F-flutemetamol retention was observed in the brain. But, ^18^F-flutemetabmol did not show a outstanding advantage in APPswe-PS1dE9 and Tg2576 mice.

However, transgenic mice with various genetic backgrounds have been related with different pathologies, which make it difficult to interpret the overlapping study results [6]. Therefore, comparisons between β-amyloid imaging regarding AD mouse have to be accomplished with some caution as brain sizes and anatomic landmarks of target VOIs greatly affect accurate PET signal quantification. Until now, there has been no antecedent report comparing between ^18^F-flutemetamol and ^18^F-florbetaben images in an AD mouse model, so comparative conclusions draw special interest.

Herein, we tested a recently developed APPsw mouse model (C57BL/6-Tg(NSE-hAPPsw)Korl) enhancing expressing Swedish double mutation form of human APP (K670 N, M671L) under regulation of the neuron specific enolase (NSE) promoter. For this mouse model, there has been no attempt regarding its application for the evaluation of new Aβ imaging ligands. Hence, we performed a small animal study conducting direct comparisons between two ^18^F labeled Aβ PET tracers, ^18^F-flutemetamol and ^18^F-florbetaben in (C57BL/6-Tg(NSE-hAPPsw)Korl) mouse model in terms of following aspects: the ability to discriminate a transgenic from a control mouse, intensity of uptake and distribution pattern in visual images, difference of static ratio and kinetic parameters, bio-distribution and correlation with neuropathologic findings.

## Methods

### Animals

Experiments were conducted with 7 APPsw transgenic mice (genetic background C57BL/6-Tg(NSE-hAPPsw)Korl) augmenting human APP with the Swedish double mutation (K670N, M671L) under regulation of the NSE promoter. As controls, 7 littermates with the corresponding genetic background, C57BL/6J, were used. Age and sex were matched between the two groups (mean age and mean weight: 18 weeks and 24.84 ± 1.01 g for APPsw mice and 18 weeks and 29.20 ± 3.49 g for C57BL/6 J control mice). The mice used in the study were donated from the Division of Laboratory Animal Resources, Korea FDA (Food and Drug safety administration, National Institute of Toxicological Research, registration number: KNL-HYD-TG0615). Details on number of animals per study group, sex, mean age and mean body weight are summarized in Table 1. Two mice from each AD and control group were sacrificed for pathology at 18 weeks and correlated with imaging. The remaining mice were sacrificed for pathology at 48 weeks. Animal experiments were conducted with the approval of the institutional animal care committee (IRB number: LML 16-970, Dong-A university, Busan, Korea).

**Table 1:** Basic characteristics of AD transgenic and control mouse model

ID	AD transgenic			Control		
	Age (weeks)	Sex	Weight (g)	Age (weeks)	Sex	Weight (g)
1	18	Male	23.41	18	Male	27.20
2	18	Male	26.34	18	Male	27.68
3	18	Male	25.12	18	Male	31.32
4	18	Male	24.16	18	Male	26.27
5	18	Male	24.12	18	Male	36.26
6	18	Male	25.68	18	Male	27.74
7	18	Male	25.11	18	Male	27.99
Mean ± SD	18	Male	24.84 ± 4.8	18	Male	29.20 ± 3.4

### PET/CT imaging

Seven transgenic and 7 control mice underwent sequential PET imaging for direct comparison of the two tracers (total 28 scans). The time interval between ^18^F-florbetaben and ^18^F-flutemetamol PET imaging was 2–3 days. Inhalation anesthesia was maintained by 3.5 L/min oxygen and 0.6–2% isoflurane, 15 min prior to scanning. The body temperature was kept at 37 °C with a temperature-controlled heating pad, and the respiratory rate stayed at 80–100/min. Small animal PET data was acquired with a nanoscan PET scanner (Mediso Medical Imaging Systems, USA). After the induction of anesthesia, the animals were positioned with their heads in the center of the field of view and were fixed in the PET scanner in the prone head first position (HFP). At the beginning of the PET scanning procedure, computed tomography (CT) scans were acquired for attenuation correction and anatomical reference (50 kVp, 250 mA). Next, simultaneous with an i.v. injection of 8.51 MBq (0.23 mCi) of ^18^F-flutemetamol or ^18^F-florbetaben, a 90-min dynamic emission scan was started. Dynamic acquisition was performed in the 3D list mode for 90 min. The emission data were normalized and corrected for decay and dead time. The sinograms were reconstructed with FBP (filtered back-projection using a ramp filter with a cut-off at the Nyquist frequency). Static images and dynamic images with 20 imaging frames were generated.

### Radiosynthesis

The radiosynthesis of ^18^F-florbetaben (4-ethoxy)phenyl]vinyl}-N-methylaniline, commercial name: Neuraceq) was performed using an auto-synthesizer according to the protocol of Piramal Enterprises Ltd. The radiochemical purity was > 99%, as determined by analytical HPLC. The radiochemical yield averaged 45% (decay-corrected) at the end of synthesis (EOS) based on ^18^F-fluorine. The specific activity averaged 774 GBq/umol at the EOS. The commercial products were purchased from the company (Duchem Bio, South Korea). The radiosynthesis of ^18^F-flutemetamol (6-Benzothiazolol, 2-[3-(^18^F) fluoro-4-(methylamino) phenyl], commercial name: Visamyl) was performed to using an auto-synthesizer according to the protocol of GE Healthcare. The radiochemical purity was > 96% as determined by analytical HPLC. The radiochemical yield averaged 27% (decay-corrected) at the end of synthesis (EOS) based on ^18^F-fluorine. The specific activity averaged 1862 GBq/umol at the EOS. The commercial products were purchased from the company (Carecamp Co., Ltd., South Korea).

### Analysis of PET data

PET data was analyzed with the fusion toolbox embedded in PMOD version 3.7.0 software (PMOD Technologies, Zurich, Switzerland). The CT image was thresholded at 2/3 of the maximal value (approximately 1340 Hounsfield units), and the skull image was obtained. For the shape of an atlas to properly fit with the skull CT, the thresholded CT image was manually fused with the magnetic resonance brain template, called M.Mirrione. Then, the transformation information was saved in a MAT-file format. Using the Initialize/Match function of the fusion toolbox, the PET image was re-sliced to match M.Mirrione template [7]. Then, the transformation information between the thresholded CT and the mouse magnetic resonance template was loaded on re-sliced PET image. Then, the re-sliced PET image was co-registered manually using the shift, rotate and scale functions and normalized to the mouse MR brain template (M. Mirrione) [7, 8]. The final co-registered PET image was masked with the M. Mirrione brain mask. The corresponding template and mask files can be found in the resources/usertemplates directory embedded in PMOD version 3.7.0 software (Pmod Technologies, Zurich, Switzerland). The same step was applied to all frames of dynamic data.

**Fig. 1:**
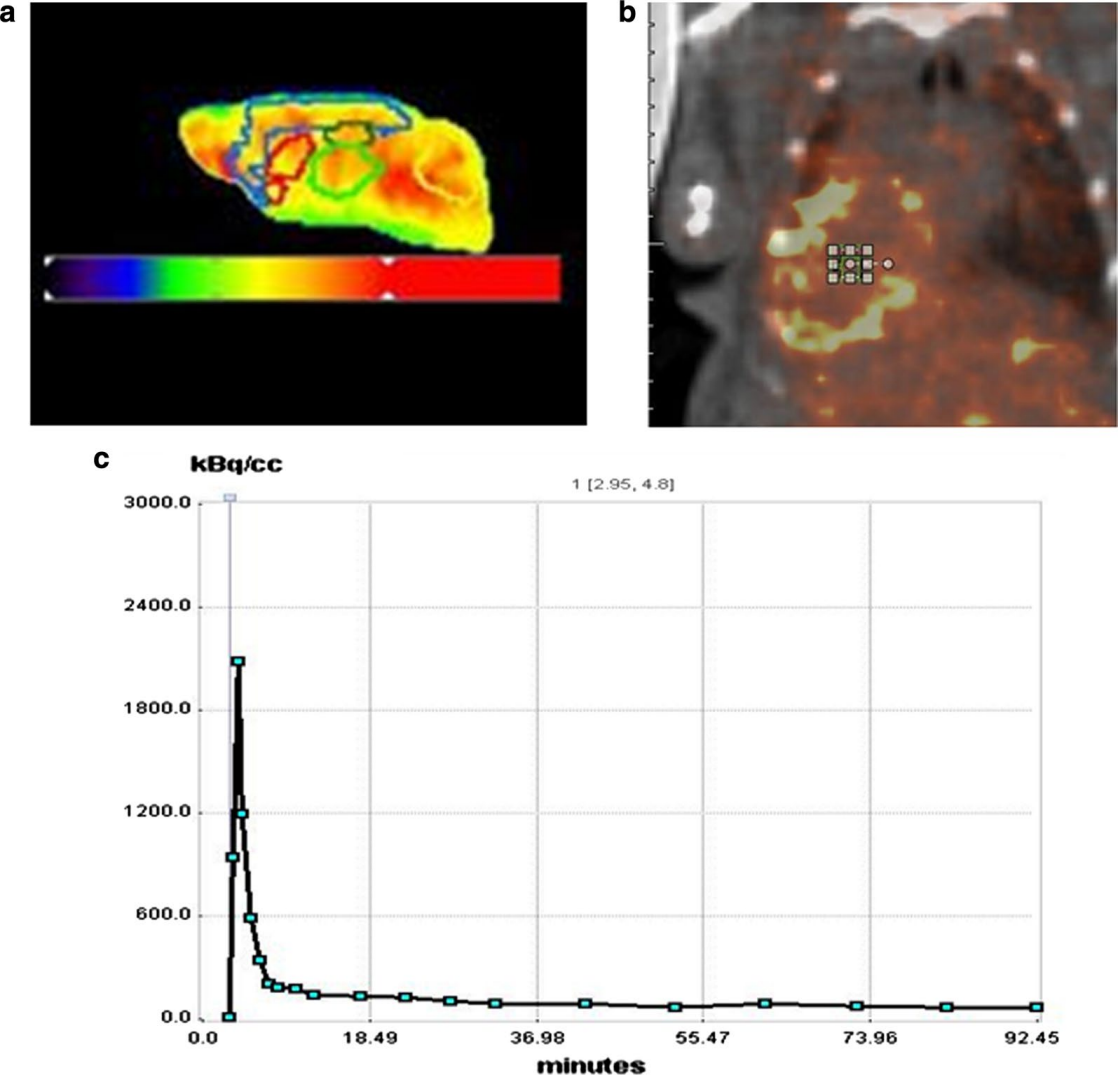
**a** Volume of interest (VOI) of mouse brain. VOI was drawn under guidance of the PMOD embedded mouse brain atlas (Mouse (M. Mirrione)-T2 MRI atlas) to cover the cortex (Cor: blue), right hippocampus (Rhip: dark green), left hippocampus (Lhip: dark green), thalamus (Thal: light green), right striatum (Rstr: red), left striatum (Lstr: red) and the cerebellum (Crbl: yellow), **b** volume of interest in blood input area, **c** time activity curve of blood input area

Volumes of interest (VOIs) of embedded mouse brains are presented in Fig. 1. The areas of the VOIs are the cortex (Cor), right hippocampus (Rhip), left hippocampus (Lhip), thalamus (Thal), right striatum (Rstr), left striatum (Lstr) and the cerebellum (Crbl). To investigate the difference between ^18^F-florbetaben and ^18^F-flutemetamol images, different images using the image algebra option embedded in PMOD fusion tool (version 3.7.0; Pmod Technologies, Zurich, Switzerland) were created. For the analysis of static PET image, the standardized uptake value (SUV) and the standardized uptake value ratio (SUVR) between the cortex and cerebellum was calculated with a VOI based method.

To determine the optimal compartment model for amyloid specific tracers, the 2 tissue compartment model from previous studies was used [9–11]. For the 2 tissue compartment model analysis with the Image Derived Input Function (IDIF) method, 1 mm^3^ volumes of interest (VOIs) were drawn on the center of the left ventricle on the initial time frame image.

### Pathology

#### Sample preparation

The animals were deeply anesthetized with zoletil and xylazine and were sacrificed by intra-cardiac perfusion with 4% paraformaldehyde (pH 7.4). The brains were embedded in paraffin wax for 48 h. The tissue samples were serially sectioned at a thickness of 10 µm on a rotary microtome for immuno-histochemical analysis.

#### Thioflavin S staining

The sections were deparaffinated and rehydrated before staining. The sections were incubated in a 1% (1 g per 100 ml water) thioflavin S (TfS, T1892, Sigma Aldrich, St. Louis, MI, USA) solution for 30 min. The sections were washed with water three times for 2 min, 80% ethanol for 6 min, washed again with water and cover slip mounted with VectaShield as the mounting medium. The slides were stored for 4 °C. The sections were washed with water three times for 2 min and with 80% ethanol for fluorescence microscopy using filter sets for DAPI and GFP. The DAPI (contained in the mounting medium) fluorescence was used by the scanner to set the optical focus, and the GFP contained the specific signal of thioflavin S.

#### Immunohistochemistry for amyloid beta 40

Non-specific reactions were blocked with 3% fetal bovine serum in phosphate buffered saline (PBS) for 1 h. Slides were incubated with mouse monoclonal primary amyloid beta 40 antibody (diluted 1:150; Millipore, USA). The secondary antibody was Streptavidin Alexa fluor 594 conjugated anti-mouse IgG (1:400, Invitrogen, USA). The fluorescence was observed using Nikon-80i fluorescence microscopy using filter sets for DAPI and RFP. The DAPI (contained in the mounting medium) fluorescence was used by the scanner to set the optical focus, and the RFP contained the specific signal of amyloid beta 40.

### Bio-distribution

The ^18^F-florbetaben and ^18^F-flutemetamol binding to different brain regions and peripheral organs in AD transgenic (N = 1) and control mice (N = 1) using ex vivo gamma counting. Mice were anaesthetized with isoflurane and injected with 0.23 mCi of ^18^F-florbetaben and ^18^F-flutemetamol. The tracer was allowed to distribute for 90 min. Mice were sacrificed by cervical dislocation and the brain was rapidly removed. Then, the blood, heart, lung, liver, kidney, medulla, cerebellum, right cortex, left cortex, olfactory bulb were dissected. ^18^F-radioactivity was measured with a gamma counter.

### Statistics

For the analysis of static PET data, group comparison of SUVR and kinetic parameters were conducted with the Mann–Whitney U. A threshold of P less than 0.05 was considered significant. All statistical analyses were performed using IBM SPSS Statistics (version 20.0; SPSS) and Medcalc 16.8.4.

## Results

### Comparative overview of representative visual brain PET image

**Fig. 2:**
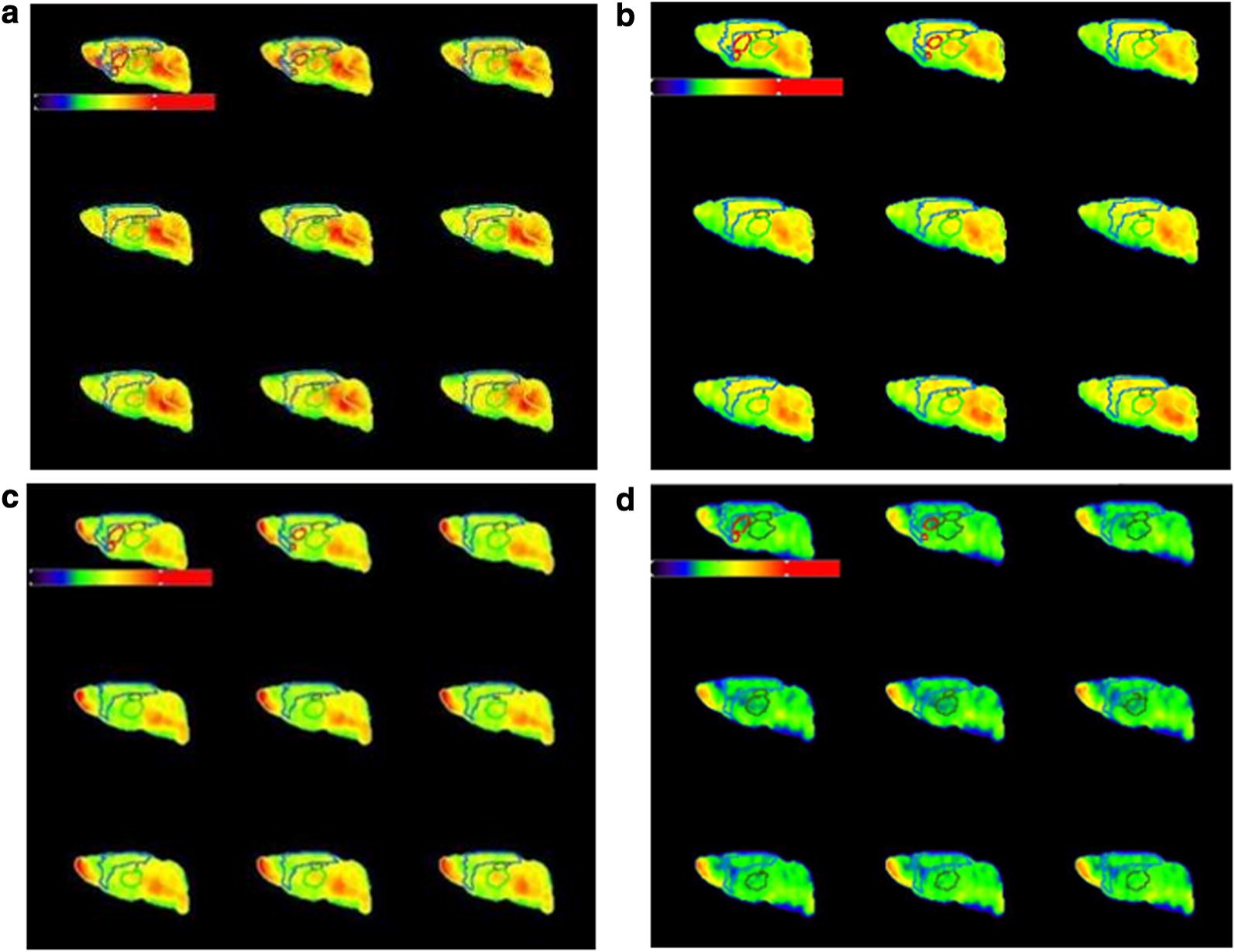
Overview of PET images sorted by study group. In both AD transgenic and control group, ^18^F-florbetaben imaging showed much higher cortical retention than did ^18^F-flutemetamol imaging. Color scale bar represents (from black to red) 0–340 percentage of injected dose per cubic centimeter in ^18^F-florbetaben image. Color scale bar represents (from black to red) 0–259 percentage of injected dose per cubic centimeter in ^18^F-flutemetamol image. **a** ^18^F-florbetaben image of AD mouse, **b** ^18^F-florbetaben image of control mouse, **c** ^18^F-flutemetamol image of AD mouse, **d** ^18^F-flutemetamol image of control mouse

A comparative overview of the representative brain PET images is presented in Fig. 2. On the ^18^F-florbetaben PET image, the AD transgenic mice showed significantly higher tracer retention in the cortical regions than did the control mice. On the ^18^F-flutemetamol PET image, the transgenic mice showed mild, focal uptake in cortical brain regions; however, higher uptake was shown in the transgenic mice than in the control mice. Overall, regardless of the AD transgenic and control group, ^18^F-florbetaben imaging showed much higher retention than did ^18^F-flutemetamol imaging. Both the AD transgenic and control groups showed high tracer retention in the cerebellum and pons than did the cortical regions.

### Difference image obtained from image algebra calculation: (^18^F-florbetaben- ^18^F-flutemetamol)

**Fig. 3:**
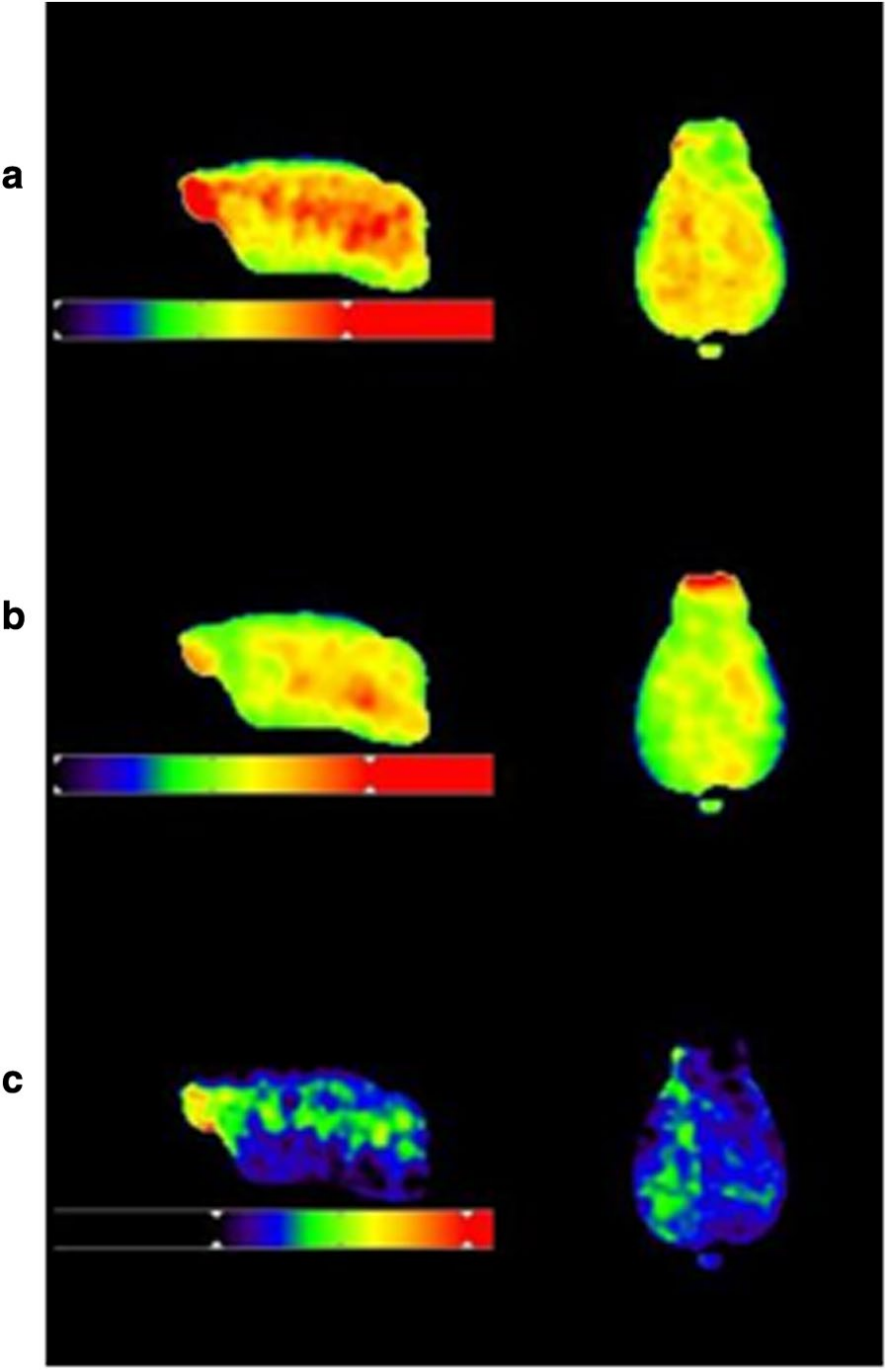
Difference between ^18^F-florbetaben and ^18^F-flutemetamol in AD transgenic group. Each image represents PET image of **a** ^18^F-florbetaben (upper column), **b** ^18^F-flutemetamol (middle column) and **c** algebra calculation (^18^F-florbetaben-^18^F-flutemetamol, lower column). In the AD transgenic group, ^18^F-florbetaben showed higher and more extensive cortical uptakes compared with ^18^F-flutemetamol. Color scale bar represents 0–340 percentage of injected dose per cubic centimeter in ^18^F-florbetaben image. Color scale bar represents 0–270 percentage of injected dose per cubic centimeter in ^18^F-flutemetamol image. Color scale bar represents 0–280 percentage of injected dose per cubic centimeter in difference image

A visual representation of comparisons of the difference between ^18^F-florbetaben and ^18^F-flutemetamol is presented in Fig. 3. In the AD transgenic group, ^18^F-florbetaben showed higher and more extensive cortical uptakes compared with ^18^F-flutemetamol.

### Comparative overview of representative visual whole body PET image

**Fig. 4:**
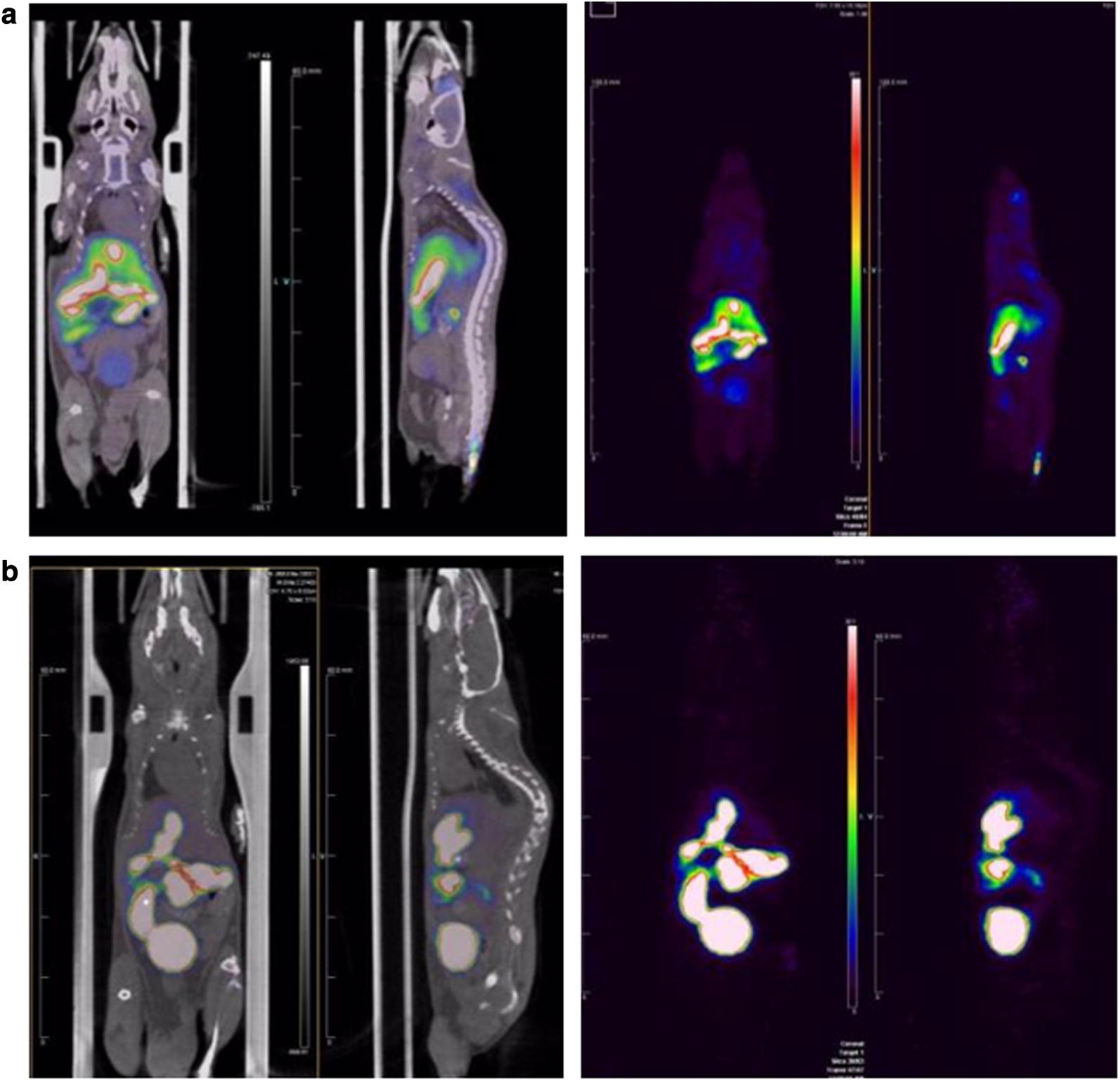
Overview of representative whole body PET images of **a** ^18^F-florbetaben and **b** ^18^F-flutemetamol in AD transgenic mouse. ^18^F-flutemetamol PET imaging showed much more intense uptake in the bowel and bladder than did ^18^F-florbetaben. Each row represents a representative PET image of the study group in sagittal view (middle column) and axial view (right column). Color scale bar represents (from black to white) 0–30% ID/g (percentage of injected dose per g) in both ^18^F-florbetaben and ^18^F-flutemetamol image

Figure 4 provides a comparative overview of the representative whole body PET images. The color bar of the PET image was adjusted to (0–30% ID/g) to optimize for visualization of the peripheral organ uptakes. ^18^F-flutemetamol PET imaging showed much more intense uptake in the bowel and bladder than did ^18^F-florbetaben.

### Bio-distribution

**Fig. 5:**
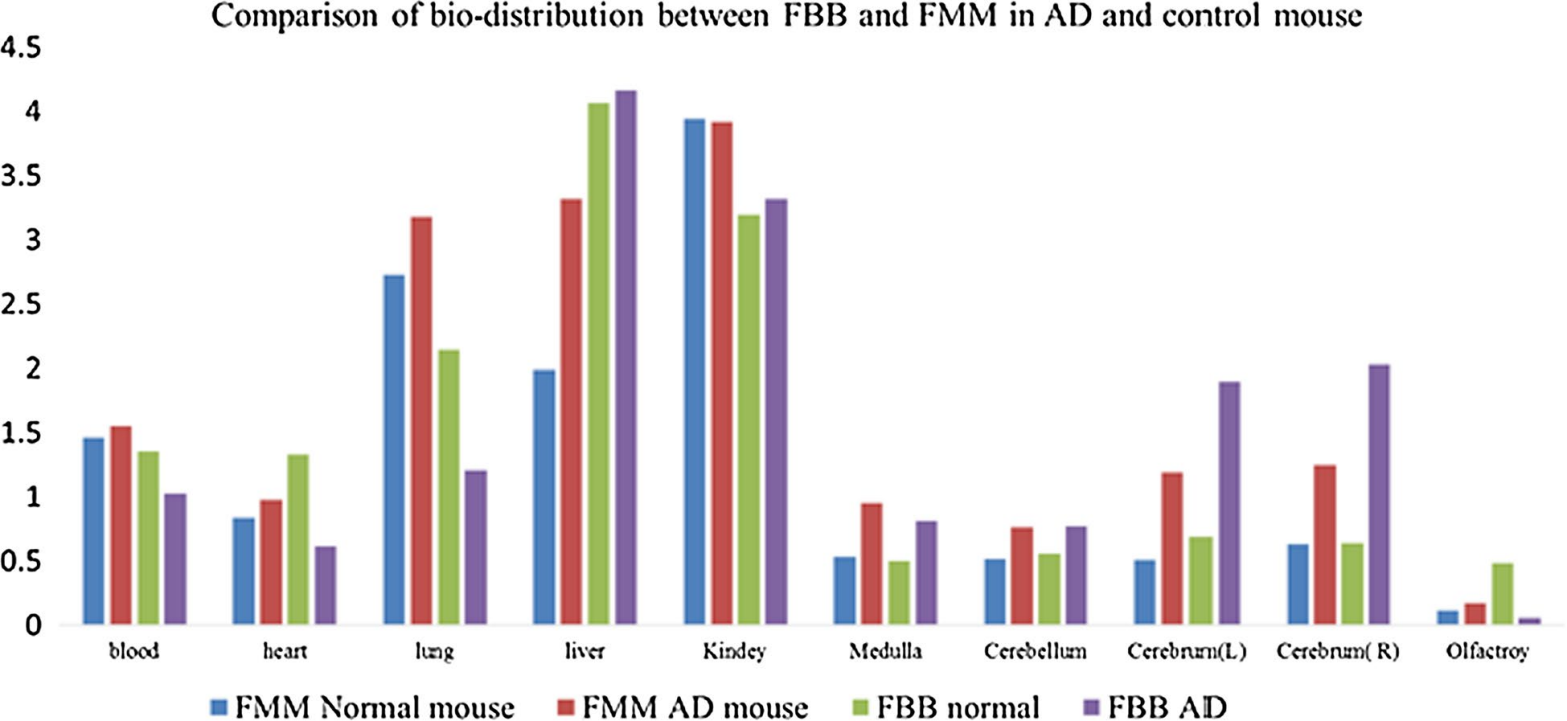
Comparison of Ex vivo bio-distribution between ^18^F-florbetaben and ^18^F-flutemetamol in AD and control mouse. ^18^F-florbetaben showed higher radioactivity in the cortex than did ^18^F-flutemetamol. In contrast, ^18^F-flutemetamol showed higher radioactivity in the kidney, lung, blood and heart

Bio-distribution data of both ^18^F-florbetaben and ^18^F-flutemetamol are presented in Fig. 5. The highest radioactivity in the brain was measured in the cortex, followed by the medulla and cerebellum. ^18^F-florbetaben (Rt. cortex: 1.39 ID/g (%), Lt. cortex: 1.209 ID/g (%)) showed higher absolute differences between AD transgenic mice and control mice than did ^18^F-flutemetamol (Rt. cortex: 0.619 ID/g (%), Lt. cortex: 0.608 ID/g (%)). In AD transgenic mouse, ^18^F-florbetaben showed higher uptake in the cortex than did ^18^F-flutemetamol. In addition, for both ^18^F-florbetaben and ^18^F-flutemetamol, the right cortex in the AD mouse showed higher uptake, showing right side laterality. In terms of the visceral distribution of ^18^F-florbetaben, the highest radioactivity was measured in the liver, followed by the kidney, lung, blood and heart in both transgenic and control mice. In terms of visceral distribution of ^18^F-flutemetamol, the highest radioactivity was measured in the kidney, followed by the liver, lung, blood and heart in transgenic mice. In control mice, the highest radioactivity was measured in the kidney, followed by the lung, liver, blood and heart. Comparative analysis of ^18^F-florbetaben and ^18^F-flutemetamol biodistribution revealed that ^18^F-florbetaben imaging showed higher radioactivity in the cortex than did ^18^F-flutemetamol. In contrast, ^18^F-flutemetamol showed higher radioactivity in the kidney, lung, blood and heart, although the liver showed higher radioactivity with ^18^F-florbetaben. In contrast with the imaging findings, the bio-distribution data showed higher uptake in the cortex than in the cerebellum.

### SUVmean and SUVR based analysis of static PET image

The SUVmean and SUVR values of PET images in both the AD transgenic and control groups are presented in Tables 2, 3. The mean SUVmean values of the ^18^F-florbetaben images in the AD and control mice were 0.804 and 0.699, respectively. In contrast, the mean SUVmean values of the ^18^F-flutemetamol images in the AD and control mice were 0.332 and 0.297, respectively. The mean SUVR values of the ^18^F-florbetaben images in the AD and control mice were 0.926 and 0.829, respectively. In contrast, the mean SUVR values of the ^18^F-flutemetamol images in the AD and control mice were 0.854 and 0.687, respectively. On both the ^18^F-florbetaben and ^18^F-flutemetamol scans, the mean SUVmean and SUVR values of the AD transgenic group showed higher values than those of the control group. The mean SUVmean and SUVR of ^18^F-florbetaben showed higher values than those of ^18^F-flutemetamol in the AD transgenic and control groups, respectively. The mean of the differences in the SUVmean between the AD and control group was 0.106 for ^18^F-florbetaben and 0.03 for ^18^F-flutemetamol.

**Table 2:** SUVR values of ^18^F-florbetaben image in both AD transgenic and control group

Group	ID	Cor	Rhip	Lhip	Thala	Rstr	Lstr
(a) *SUVR values of* ^18^*F-florbetaben image in AD transgenic group*							
AD	1	0.879	0.912	0.941	1.017	0.917	0.979
	2	0.958	0.843	0.808	0.932	0.883	0.878
	3	0.929	0.988	1.004	1.083	1.015	1.014
	4	0.838	0.955	1.009	1.084	0.978	1.000
	5	0.966	0.996	0.966	1.030	0.870	0.856
	6	0.919	1.121	1.133	1.258	1.161	1.187
	7	0.994	1.074	1.133	1.216	1.071	1.144
Average		0.926	0.984	0.999	1.089	0.985	1.008
SD		0.054	0.094	0.113	0.114	0.106	0.123

(b) *SUVR values of* ^18^*F-florbetaben image in control group*							
Control	1	0.663	0.901	0.966	1.023	0.948	0.956
	2	0.852	0.964	0.957	1.025	0.932	0.962
	3	0.872	0.944	1.006	1.098	1.016	1.035
	4	0.854	1.780	1.775	1.997	1.762	1.752
	5	0.876	0.894	1.003	1.054	0.960	0.988
	6	0.822	1.000	0.989	1.085	1.000	1.012
	7	0.862	0.918	0.972	1.084	0.966	1.007
Average		0.829	1.057	1.095	1.195	1.084	1.102
SD		0.070	0.297	0.278	0.329	0.278	0.267

*Cor* cortex, *Rhip* Rt. hippocampus, *Lhip* Lt. hippocampus, *Thala* Thalamus, *Rstr* Rt. striatum, *Lstr* Lt. striatum

**Table 3:** SUVR values of ^18^F-flutemetamol image in both AD transgenic and control group

Group	ID	Cor	Rhip	Lhip	Thala	Rstr	Lstr
(a) *SUVR values of *^18^*F-flutemetamol image in AD transgenic group*							
AD	1	0.872	0.997	1.037	1.116	1.038	1.094
	2	0.849	0.913	0.882	1.086	0.978	0.974
	3	0.922	0.991	1.064	1.147	1.035	1.144
	4	0.782	0.922	0.939	1.016	0.897	0.889
	5	0.814	0.989	0.950	1.129	0.995	0.899
	6	0.946	0.989	1.003	1.134	1.035	1.123
	7	0.792	0.880	0.933	0.933	0.791	0.837
Average		0.854	0.954	0.973	1.080	0.967	0.994
SD		0.063	0.048	0.064	0.078	0.092	0.125

(b) *SUVR values of* ^18^*F-flutemetamol image in control group*							
Control	1	0.589	0.623	0.633	0.720	0.650	0.638
	2	0.674	0.718	0.784	0.839	0.738	0.779
	3	0.765	0.839	0.862	0.940	0.814	0.829
	4	0.684	0.743	0.773	0.837	0.764	0.762
	5	0.706	0.904	0.912	1.015	0.923	0.950
	6	0.685	0.822	0.848	0.873	0.752	0.771
	7	0.703	0.736	0.864	0.928	0.818	0.850
Average		0.687	0.769	0.811	0.879	0.780	0.797
SD		0.053	0.093	0.092	0.094	0.084	0.095

*Cor* cortex, *Rhip* Rt. hippocampus, *Lhip* Lt. hippocampus, *Thala* Thalamus, *Rstr* Rt. striatum, *Lstr* Lt. striatum

#### Statistical analysis of static PET data: AD vs. control

The quantitative parameters of the static PET images (SUVR) in the AD transgenic and control groups were tested. On the ^18^F-florbetaben images, the AD transgenic group showed significantly higher SUVR values (p = 0.011) than did the control group. On the ^18^F-flutemetamol images, the AD group showed significantly higher SUVR values (p = 0.001) than did the control group. Moreover, on the ^18^F-flutemetamol images, the AD group showed significantly higher SUVR values than did the control group in all brain areas. However, on the ^18^F-florbetaben images, the AD group showed significantly higher SUVR values than did the control group only in the cortex.

#### Statistical analysis of static PET data: ^18^F-florbetaben vs. ^18^F-flutemetamol

The quantitative parameters of the static PET images (SUVR) between the ^18^F-florbetaben and ^18^F-flutemetamol groups are presented in Table 4. The significant differences of the SUVR (cortex/cerebellum) between the scans of the two tracers in each AD and control group were compared. ^18^F-florbetaben presented a higher SUVR value in the cortex than did ^18^F-flutemetamol in both the AD (p = 0.049) and control groups (p = 0.017).

**Table 4:** Comparison of SUVR values between ^18^F-florbetaben and ^18^F-flutemetamol in AD transgenic group

Group	Cor	Rhip	Lhip	Thala	Rstr	Lstr
^18^F-flutemetamol	0.851 ± 0.063	0.950 ± 0.051	0.972 ± 0.064	1.081 ± 0.080	0.970 ± 0.092	0.9910 ± .092
^18^F-florbetaben	0.931 ± 0.050	0.980 ± 0.090	1.000 ± 0.110	1.090 ± 0.113	0.991 ± 0.113	1.010 ± 0.124
*p* value	0.049**	0.805	0.456	0.805	1.000	0.805

*Cor* cortex, *Rhip* Rt. hippocampus, *Lhip* Lt. hippocampus, *Thala* Thalamus, *Rstr* Rt. striatum, *Lstr* Lt. striatum, ** p < 0.05 considered as significant

### Quantitative compartment model dynamic analysis of ^18^F-florbetaben and ^18^F-flutemetamol image

#### Statistical analysis of dynamic PET data: AD versus. control

In the ^18^F-florbetaben group, there was a significant difference in the K1 (p = 0.011) and k4 (p = 0.017) parameters between the AD transgenic and control groups. However, in the ^18^F-flutemetamol group, there was no significant difference in K1, k2, k3, k4, K1/k2, or k3/k4 between the AD transgenic and control groups.

#### Statistical analysis of dynamic PET data: ^18^F-florbetaben and ^18^F-flutemetamol

In the AD transgenic group, there were significant differences of K1 (Table 5), k4 (Table 6), and K1/k2 between ^18^F-florbetaben and ^18^F-flutemetamol. In the control group, there were differences in k3 and k3/k4 between ^18^F-florbetaben and ^18^F-flutemetamol.

**Table 5:** Comparison of K1 values (2 compartment model) between ^18^F-florbetaben and ^18^F-flutemetamol in AD transgenic group

Group	Rstr	Lstr	Cor	Rhip	Lhip	Thal	Crbl
^18^F-flutemetamol							
Mean + SD	7.380 ± 1.032	7.331 ± 1.103	6.861 ± 1.331	6.972 ± 1.303	7.3 ± 0.900	7.841 ± 0.240	7.400 ± 1.410
Median (IQR)	7.98 (6.95–8)	8 (6.84–8)	7.52 (5.85–7.95)	7.29 (6.38–8)	7.92 (6.31–8)	7.95 (7.61–8)	8 (7.66–8)
^18^F-florbetaben							
Mean + SD	4.991 ± 3.091	4.581 ± 2.960	3.561 ± 2.160	4.182 ± 2.160	4.802 ± 2.160	4.891 ± 2.160	5.060 ± 2.160
Median (IQR)	4.9 (1–8)	4.51 (1–8)	3.92 (1–4.63)	3.84 (1–7.63)	4.78 (1–7.95)	4.88 (1.18–8)	5.84 (1.65–7.61)
p-value	0.128	0.053	0.011**	0.073	0.128	0.038**	0.073

*Cor* cortex, *Crbl* cerebellum, *Rhip* Rt. hippocampus, *Lhip* Lt. hippocampus, *Thala* Thalamus, *Rstr* Rt. striatum, *Lstr* Lt. striatum, ** p < 0.05 considered as significant

**Table 6:** Comparison of k4 values (2 compartment model) between ^18^F-florbetabenand ^18^F-flutemetamol in AD transgenic group

Group	Rstr	Lstr	Cor	Rhip	Lhip	Thal	Crbl
^18^F-flutemetamol							
Mean + SD	2.281 ± 3.510	1.622 ± 2.793	2.950 ± 3.210	4.551 ± 3.982	2.432 ± 3.982	1.281 ± 3.982	2.403 ± 3.982
Median (IQR)	0.331 (0.18–6.77)	0.561 (0.35–1.06)	1.752 (0.37–7.23)	7.460 (0.32–7.87)	0.340 (0.13–7.71)	0.273 (0.11–0.44)	0.374 (0–7.28)
^18^F-florbetaben							
Mean + SD	0.713 ± 1.010	1.327 ± 2.290	0.161 ± 0.150	0.321 ± 0.331	0.902 ± 1.880	2.190 ± 3.102	0.140 ± 0.141
Median (IQR)	0.312 (0.22–0.92)	0.283 (0.25–2.03)	0.240 (0–0.29)	0.282 (0–0.41)	0.281 (0–0.35)	0.383 (0.21–6.15)	0.110 (0–0.3)
p-value	0.902	0.383	0.017**	0.053	0.383	0.535	0.165

*Cor* cortex, *Crbl* cerebellum, *Rhip* Rt. hippocampus, *Lhip* Lt. hippocampus, *Thala* Thalamus, *Rstr* Rt. striatum, *Lstr* Lt. striatum, ** p < 0.05 considered as significant

#### Difference in the time-activity curve between ^18^F-florbetaben and ^18^F-flutemetamol

**Fig. 6:**
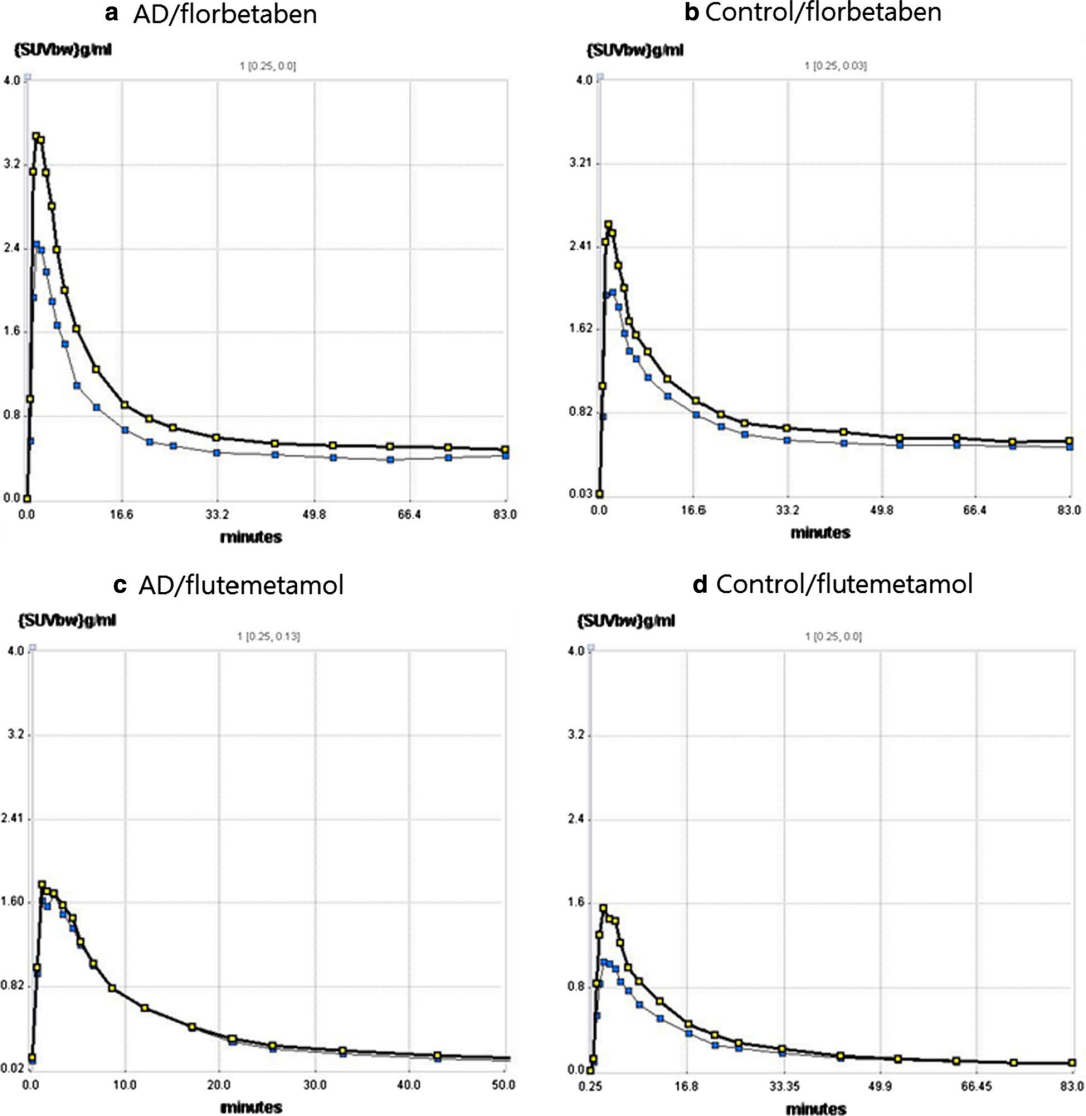
Dynamic PET time activity curves of the cortex-VOI and the cerebellum-VOI. Time-activity curves of **a** ^18^F-florbetaben in AD mouse, **b** ^18^F-florbetaben in control mouse, **c** ^18^F-flutemetamol in AD mouse, **d** ^18^F-flutemetamol in control mouse, were illustrated. Values are SUVbw (g/ml) for a cortex VOI (blue line) and a cerebellum VOI (black line). ^18^F-florbetaben showed higher initial uptake and later retention than did ^18^F-flutemetamol. In contrast, ^18^F-flutemetamol showed lower initial upstroke and faster washout than did ^18^F-florbetaben

Dynamic PET time activity curves of the cortex-VOI and cerebellum-VOI for the two tracer images in representative AD and control mice are illustrated in Fig. 6. Visual inspection of the time-activity curves revealed that ^18^F-florbetaben showed higher initial uptake and later retention than did ^18^F-flutemetamol. In contrast, ^18^F-flutemetamol showed lower initial upstroke and faster washout than did ^18^F-florbetaben.

### Neuropathologic findings (at 18 weeks)

#### Hematoxylin and eosin (H & E) staining

**Fig. 7:**
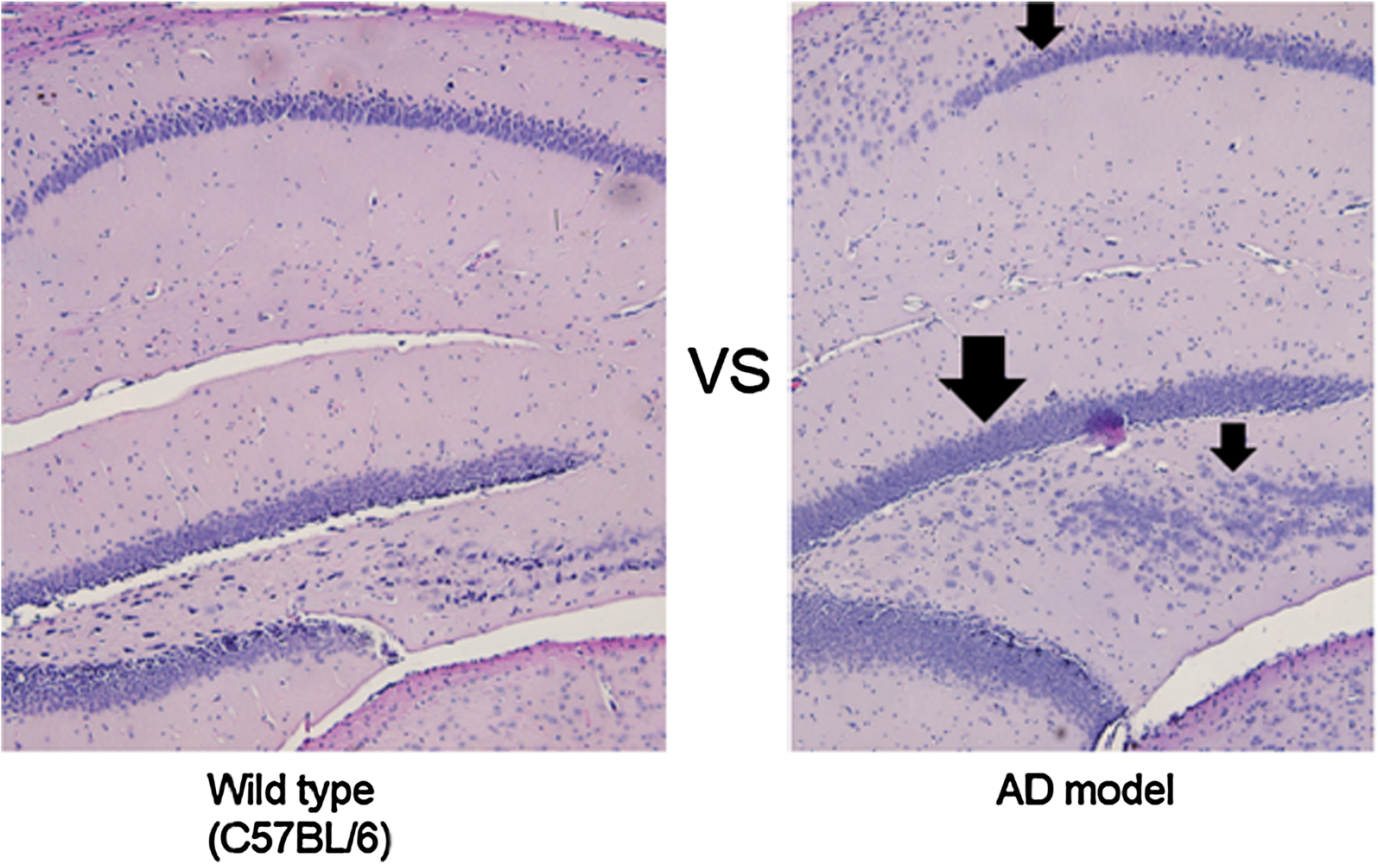
Visual comparison of the H & E staining image of hippocampus (sagittal section) between AD and wild type. Left: the zoom (100×) of the hippocampus in wild type, Right: the zoom (100×) of the hippocampus in AD mouse. AD mice show a more immature pattern as a result of disarrangement of hippocampal cell migration along the dentate gyrus of the hippocampus compared with the wild type

In Fig. 7, the AD mice show a more immature pattern as a result of disarrangement of hippocampal cell migration (pathogenic sign of AD) along the dentate gyrus of the hippocampus compared with the wild type.

#### Thioflavin S staining image

**Fig. 8:**
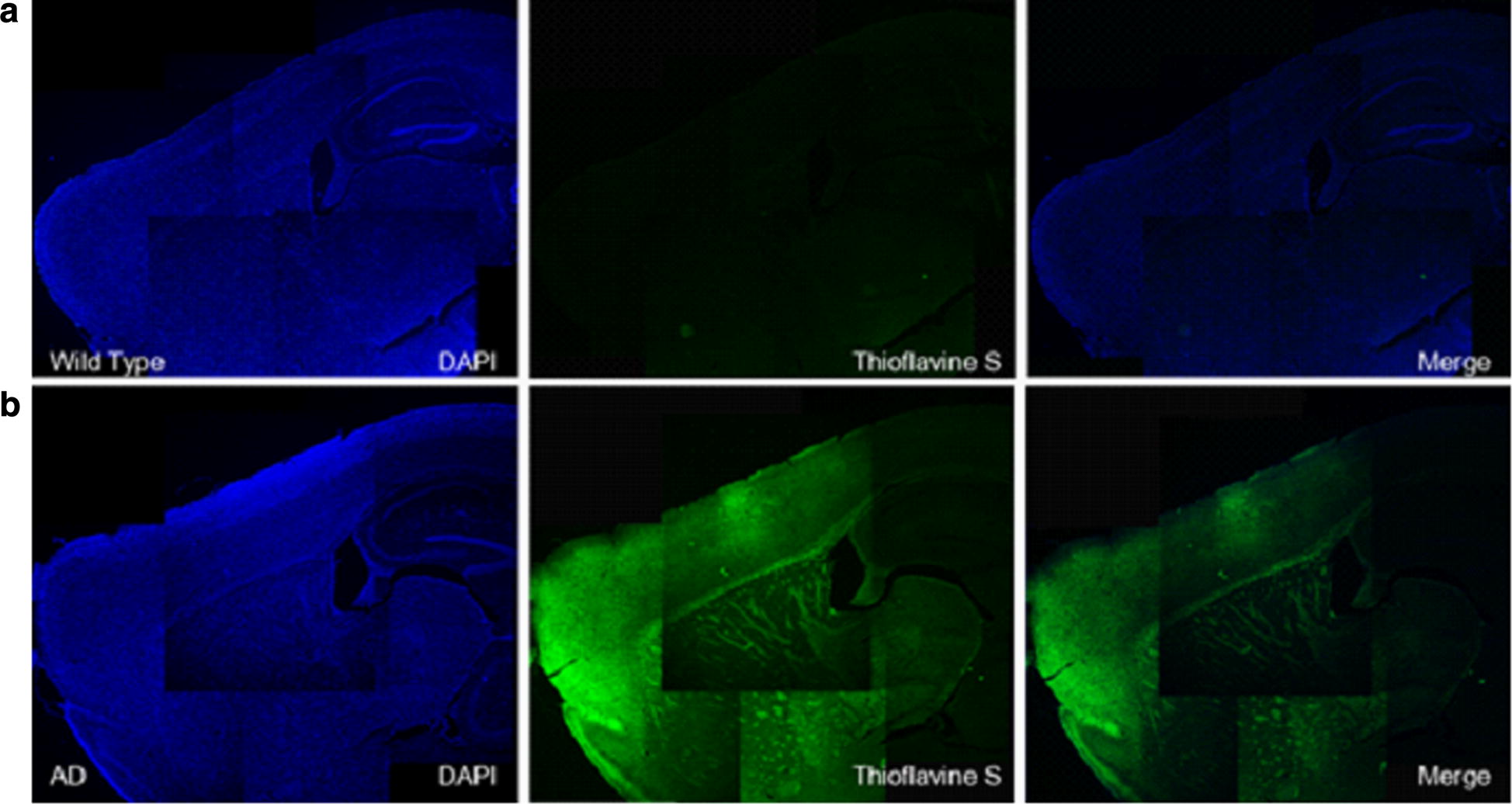
Visual overview of the thioflavin S staining images of **a** wild type mouse and **b** AD transgenic mouse. Left column shows DAPI (blue channel), middle column shows thioflavin S (green channel with specific staining signal) and right column shows merged image. Aβ deposits were found broadly in various brain regions including the cortex, hippocampus and thalamus in AD mice

On thioflavin S staining images, Aβ deposits were found broadly in various brain regions including the cortex, hippocampus and thalamus in AD mice. Thioflavin S positive plaque areas predominantly diffuse in a morphologic characteristic nature rather than in a compact nature (Fig. 8).

#### Immunohistochemistry for Aβ_40_ staining

**Fig. 9:**
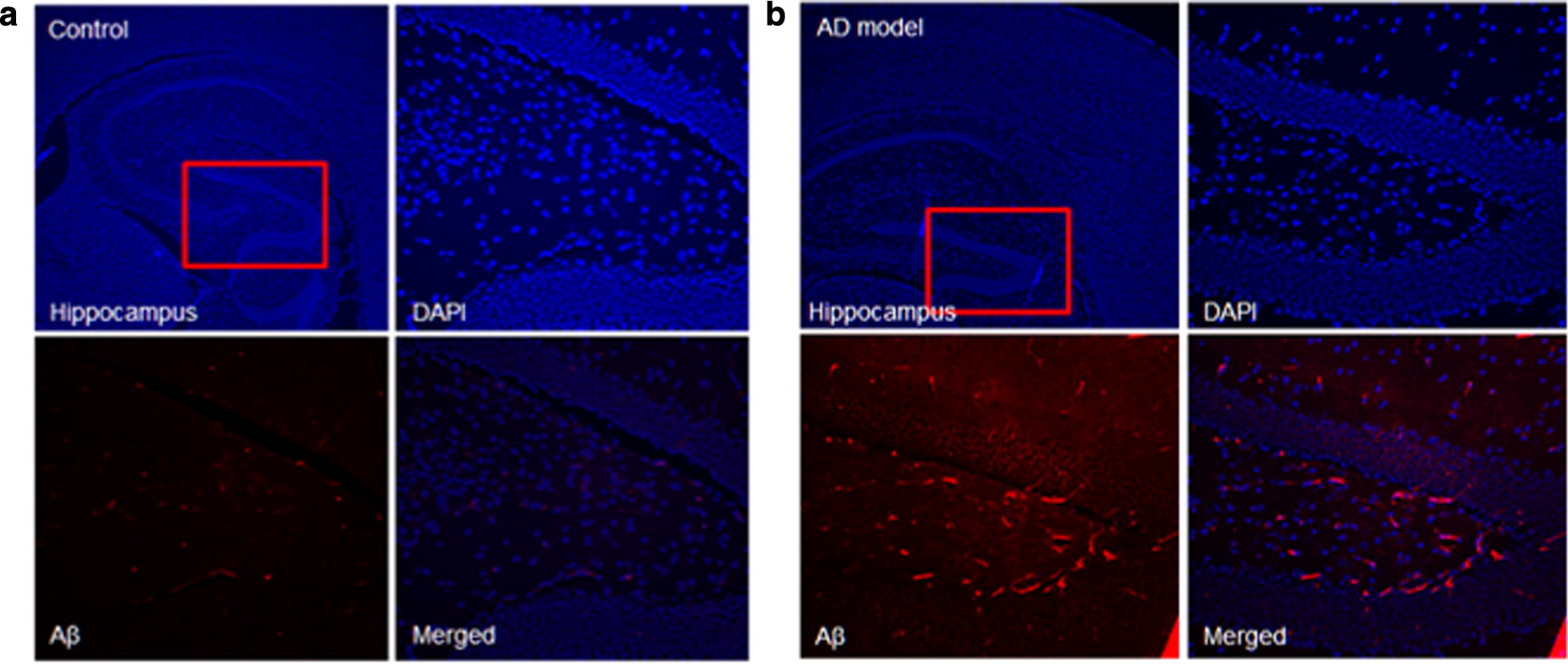
Visual overview of the Aβ_40_ staining images of hippocampus in **a** wild type and **b** AD mouse. In each group, right upper row shows DAPI (blue channel), Left lower panel shows Aβ_40_ (RFP red channel with specific staining signal) and Right column shows merged image. In wild type mouse, there was no Aβ 40 expression in the hippocampus. In contrast, the Aa40 expression of AD transgenic mouse significantly increased

**Fig. 10:**
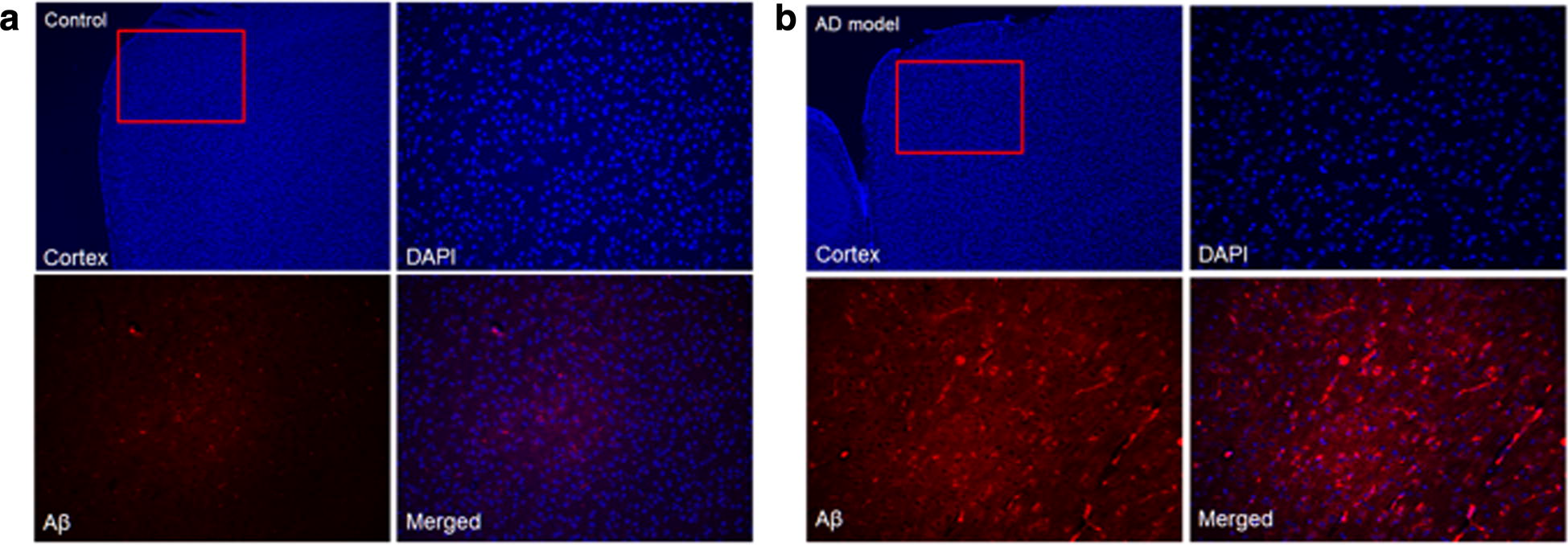
Visual overview of the Aβ_40_ staining images of cortex in **a** wild type and **b** AD mouse. In each group, right upper row shows DAPI (blue channel), Left lower panel shows Aβ_40_ (RFP red channel with specific staining signal) and Right column shows merged image. In wild type mouse, there was no Aβ 40 expression in the cortex. In contrast, the Aa40 expression of AD transgenic mouse significantly increased

The results of immunohistochemistry for Aβ_40_ staining in AD transgenic and wild type were represented in Figs. 9, 10. In wild type mouse, there was no Aβ _40_ expression in the cortex and hippocampus. In contrast, the Aa_40_ expression of AD transgenic mouse significantly increased, correlating our H & E staining findings.

### Correlation with neuropathologic findings and visual PET image

**Fig. 11:**
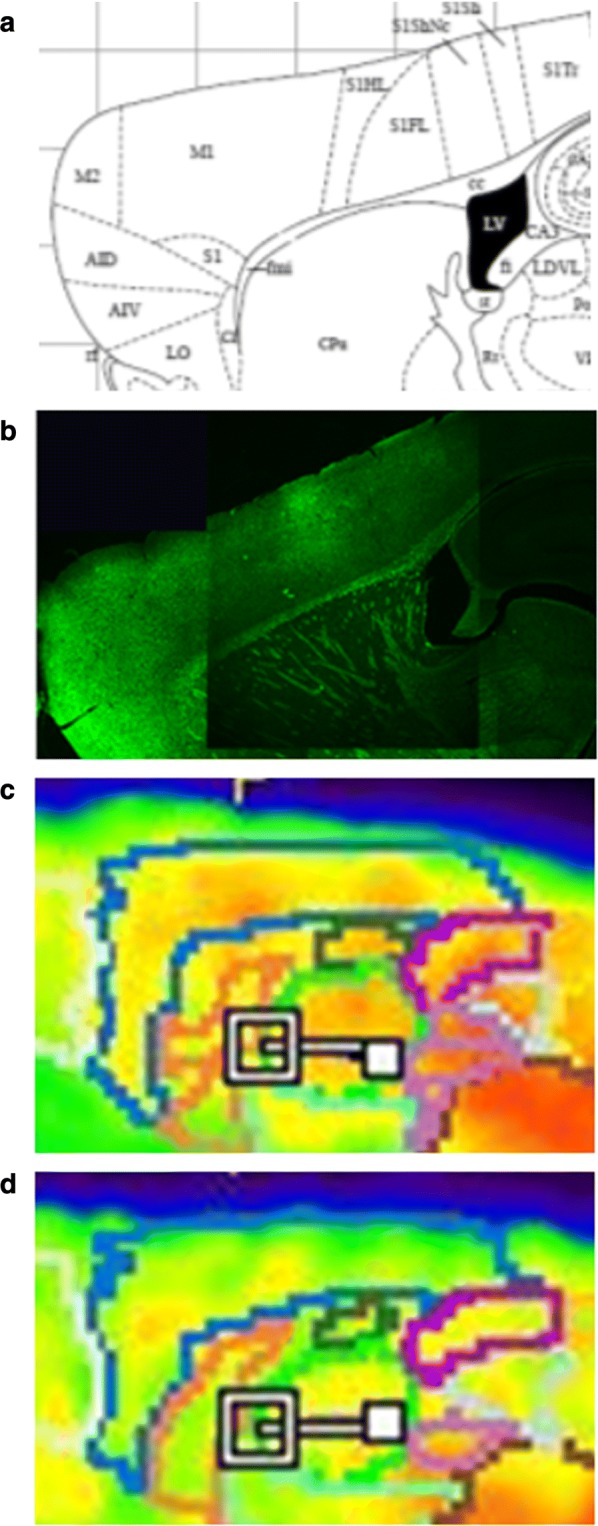
Correlation with neuropathologic finding and visual PET images. **a** Paxinos and Franklinis the Mouse Brain in stereotaxic coordinates atlas representing our pathological section, **b** Thioflavin S staining image, **c** ^18^F-florbetaben image, **d** ^18^F-flutemetamol image. The ^18^F-florbetaben PET images matched well to the thioflavin S staining image in aspects of signal intensity and spatial distribution pattern in cortical brain regions, compared with ^18^F-flutemetamol images

Finally, as shown in Fig. 11, the ^18^F-florbetaben PET images more closely correlated with the thioflavin S staining image in terms of spatial distribution pattern. However, the ^18^F-flutemetamol images revealed less prominent signal intensity and poor correlation of spatial distribution with neuropathologic plaque distribution shown in thioflavin S staining images.

### Follow-up neuropathologic findings (at 48 weeks)

**Fig. 12:**
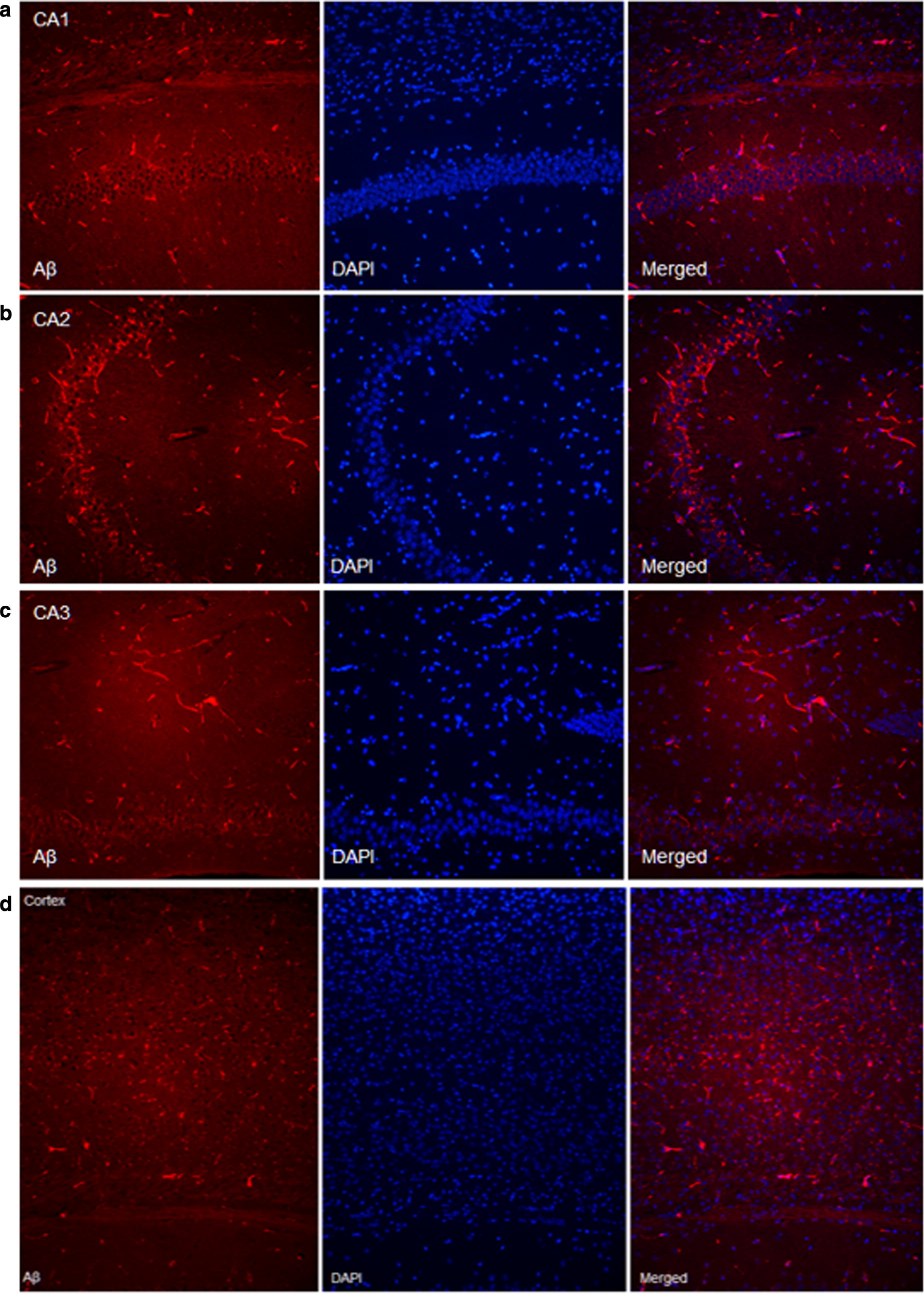
Aβ_40_ staining images of AD mouse at 48 weeks in **a** CA1, **b** CA2, **c** CA3 area of hippocampus and **d** cortex

**Fig. 13:**
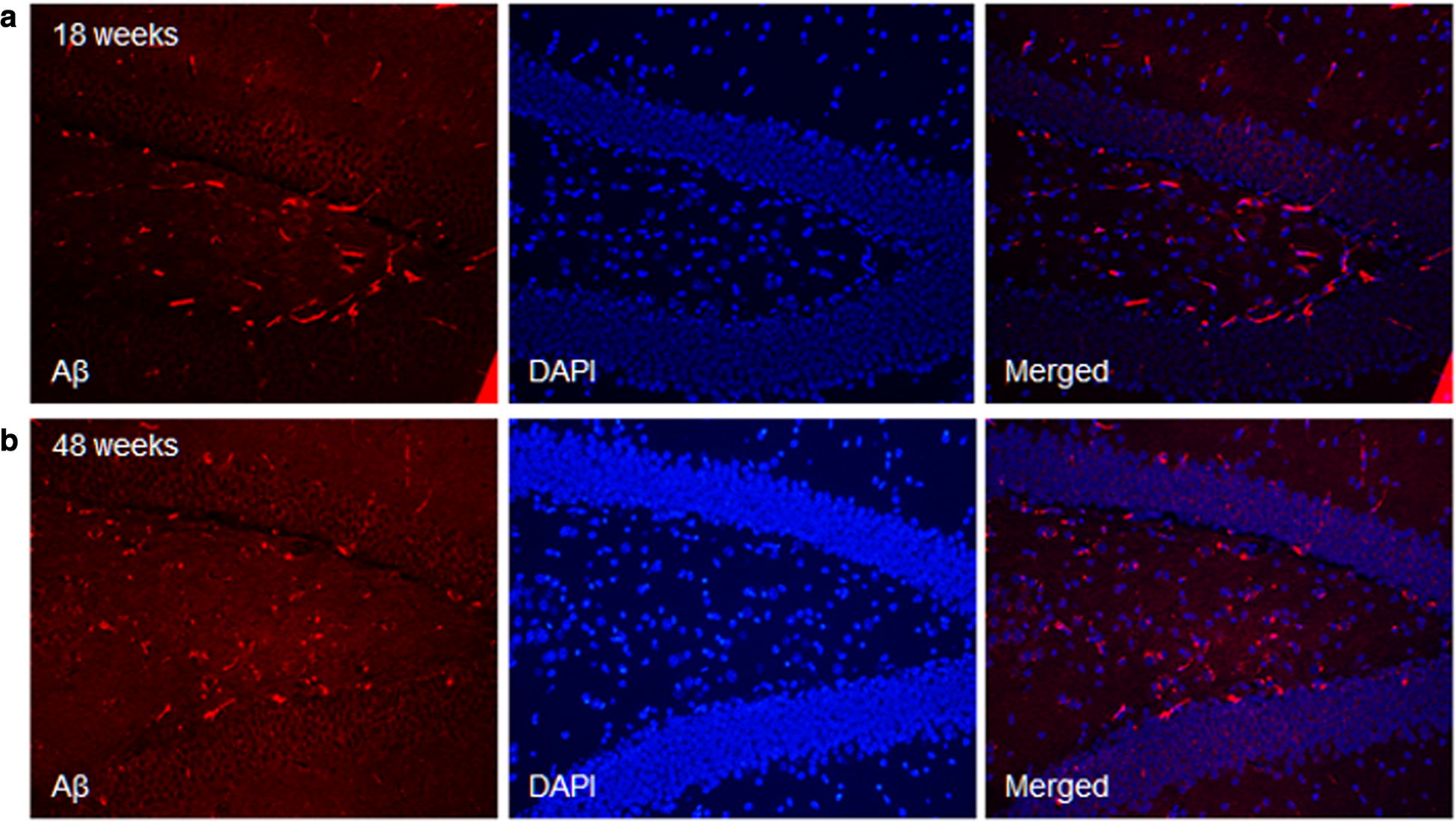
Comparison of the Aβ_40_ staining images of AD mouse in dentate gyrus of hippocampus between **a** 18 week and **b** 48 weeks. AD mice showed extensive and significantly increased Aβ40 expression in the hippocampus and cortex compared with the images at 18 weeks

The results of the follow-up immunohistochemistry for Ai_40_ staining in AD mice at 48 weeks are shown in Figs. 12, 13. At 48 weeks, AD mice showed extensive At_40_ expression in dentate gyrus of hippocampus (CA1, CA2, CA3) and cortex, compared with the images at 18 weeks.

## Discussion

In this study, ^18^F-florbetaben and ^18^F-flutemetamol images could differentiate AD and control group on visual and SUVR analysis. The ^18^F-florbetaben group presented differences in K1 and k4 kinetic parameters between AD and control groups, although ^18^F-flutemetamol did not show difference. Several differences emerged between ^18^F-florbetaben and ^18^F-flutemetamol. ^18^F-florbetaben images showed more prominent visual uptake intensity and higher SUVR than the ^18^F-flutemetamol images did. Moreover, ^18^F-florbetaben PET images more correlated well with the thioflavin S staining. However, according to bio-distribution and kinetic results, ^18^F-flutemetamol is more actively metabolized than is ^18^F-florbetaben, suggesting that ^18^F-flutemetamol has faster transport from arterial plasma into the first tissue compartment and faster dissociation from the amyloid tracer complex.

In the static analysis data, the results were grossly consistent with a previous study [12]. In another ^18^F-florbetaben PET study, the SUVR in APPswe/PS2 at 5 months was 0.95 ± 0.04, and the SUVR in APPswe/PS1G384A mice at 5 months was 0.93 [12]. The traditional SUVR method measures the radioactivity ratio of brain target regions to reference tissue during a fixed time interval after injection of the tracer [11]. This relative quantitative approach for static PET data is practical for routine clinical setting. However, due to the kinetic compartment model for reversible binding radiotracers such as ^18^F-florbetaben or ^18^F-flutemetamol, the kinetic model reflects the available binding site density and also the perfusion signal and tracer clearance to and from brain tissue [11]. In this study, the 2 tissue compartment model with IDIF method was used, and the IDIF appears to be an attractive non-invasive alternative option obviating the need for arterial cannulation, blood handling and analysis [13–15]. Furthermore, to avoid the effects of non-specific binding, we prolonged the uptake time, resulting in a longer wash-out of non-specifically bound tracer. A clinical protocol for ^18^F-florbetaben involves a 90 min uptake periods [14]. A similar protocol was used in the previous APPPS1-21 mouse cohort study, allowing a 90-min uptake time [15].

The reasons for the disparity in imaging characteristics between ^18^F-florbetaben and ^18^F-flutemetamol are related to their chemico-physiological properties. ^18^F-florbetaben and ^18^F-flutemetamol belong to different families of imaging probes. ^18^F-flutemetamol is a member of the thioflavin derivatives imaging probe family [16], and ^18^F-florbetaben belongs to a different branch of imaging probe family, the trans-stilbene derivatives [16]. Because these two tracers belong to distinct chemical families, they showed differences in binding affinity. In the bio-distribution data, ^18^F-flutemetamol showed lower brain and higher peripheral organ uptake responsible for metabolite excretion compared with ^18^F-florbetaben. These findings and kinetic parameter results suggest that ^18^F-flutemetamol is more actively metabolized than is ^18^F-florbetaben. The tracer metabolites were more polar than were the parent molecules and therefore less able to enter the brain [17]. In a preclinical study comparing the pharmacokinetic characteristics of ^18^F-flutemetamol with that of ^11^C-PiB, the metabolism of ^18^F-flutemetamol was faster than that of ^11^C-PiB [18]. This finding can be explained by the higher lipophilicity of ^18^F-flutemetamol (logPC18 = 1.7) than that of ^11^C-PiB (logPC18 = 1.2) [19]. In another study, the lipophilicity of ^18^F-florbetaben (Log Doct/PBS = 1.58) was higher than that of ^11^C-PiB (Log Doct/PBS = 1.50) [20]. These results indirectly demonstrate that the rapid metabolism of ^18^F-flutemetamol could be explained by the higher lipophilicity of ^18^F-flutemetamol (logPC18 = 1.7) than of ^18^F-florbetaben (Log Doct/PBS = 1.58).

Previous studies reported that various transgenic animal models showed differences in binding affinity with imaging tracers and this phenomenon was thought to be related with variations in plaques configurations. In this study, 18-week-old AD transgenic mice carrying NSE-controlled APPswe, C57BL/6-Tg (NSE-hAPPsw) Korl were selected due to their rapid and robust amyloid plaque development at that early age [21]. In contrast, Tg2576 mice showed late onset and slower accumulation [5]. In another previous report using APPPS1 mice co-expressing L166P mutated Presenilin 1 under the control of a neuron-specific Thy1 promoter and KM670/671NL mutated amyloid precursor protein, cortical amyloidosis was reported at the age of 6–8 weeks [22]. In APPPS1-21 mice, amyloid was known to accumulate in a 4-week and cortical microglia increased threefold from 1 to 8 months of age [22]. Hence, APPPS1 mice are good for investigating the mechanism of amyloidosis and treatment strategies because of their early onset of amyloid deposition and convenient cross-breeding with other genetically engineered mouse models.

In humans, the APPswe gene caused early presentation of familial AD [23]. In the NSE-controlled APPswe mouse model, the Swedish double mutation at the 670/671 codon in the human APP gene under the control of the NSE promoter caused increased cleavage by the beta secretase and accelerated amyloid accumulation at young age [24]. Amyloid deposition in the NSE-controlled APPswe mouse induces subsequent neuronal apoptosis through the mechanisms of the mitogen-activated protein kinase (MAPK) and c-Jun N-terminal kinase (JNK) pathway or caspase-3 pathway [25, 26]. Although the mouse model in our study was relatively young, compelling evidence from previous studies regarding the dynamics of cerebral amyloidosis using a young APP mouse model indicates that NSE-augmented APPswe mice are suitable for the neuropathological phenotype of AD [27]. Moreover, we selected only male mice to control the effect of sex. Several studies reported the effect of sex on β-amyloid accumulation and AD phenotype. Latest studies have investigated the effects of sex on hippocampal atrophy in normal aging, MCI and AD [28]. Sex could regulate the relation of amyloid positivity and cognition [28]. Also, significant sex differences in pathology of 3xTg-AD mice suggested these differences may be due to organizational actions of sex hormones during development [29].

The results of this study are in contrary to those of antecedent ^18^F-FDDNP study that presented affinity for both amyloid and neurofibrillary tangles [30]. There was no increase in cortical uptake even in 13–15-month-old Tg2576 mice, even if technical issues, such as low spatial resolution, were regarded as the reasons for this negative PET finding [30]. However, in another ^18^F-FDDNP study in triple-transgenic rats, previous partial volume effects were overcome and contrasting results were observed; prominent uptake was presented in the frontal cortex and hippocampus [31]. In an ^11^C-PiB study, old Tg2576 mice showed prominent cortical binding than did control mice [9]. Those paradoxical results were explained by the confounding effects of cortical perfusion and the low distribution of ^11^C-PiB binding sites per plaque [32]. However, following the ^11^C-PiB study with high specific activity overcame such confounding effects, so significant cortical uptake and excellent correlation between PET uptake and a pathologic amyloid burden were observed in APP23 mice compared with age-matched healthy controls [33]. Additionally, in a recent ^11^C-PiB PET study of APP/PS1 mice, an outstanding correlation can be found between imaging results and the plaque burden measure obtained ex vivo and in vitro in the same animals [34]. In a previous preclinical imaging study comparing ^18^F-florbetaben and ^11^C-PiB, which is of the same thioflavin T derivative family with ^18^F-flutemetamol, two aged AD mouse models with contrasting levels of amyloid deposition to high (APPPS 1-21) and low (BRI 1-42) target state were investigated [15]. Compared with control mice, APPPS 1-21 mice (high target state) presented prominent fibrillary amyloid accumulation in both ^11^C-PiB and ^18^F-florbetaben, but the difference of uptake between AD and control mice was higher for ^11^C-PiB than for ^18^F-florbetaben [15]. However, BRI1-42 mice (low target state) did not show enhanced tracer uptake [15]. Taking into consideration the difference in the mouse ages, our results broadly resemble their findings. Another ^18^F-florbetaben study using the same mouse cohort reported only a 14.5% difference between control and transgenic mice (5XFAD) found with ^18^F-florbetapir in comparison to a 21% difference found with ^11^C-PiB [35].

Before the interpretation of ^18^F-florbetaben or ^18^F-flutemetamol images in clinical settings, preclinical approaches can provide baseline information regarding differences in the kinetic and metabolic properties of two tracers. The visual image and SUVR of ^18^F-florbetaben showed extensive cortical uptake in the same cohorts compared with ^18^F-flutemetamol images in both the AD and control groups. On the ^18^F-flutemetamol images, high lipophilicity and fast metabolism might complicate the analysis of PET data. In this study, the metabolism and kinetics of the tracer also have a great influence on the visual uptake of the amyloid tracers. Using these points, the human amyloid image should be read in consideration of the pharmacokinetic and metabolic properties of the tracer. Therefore, preclinical imaging might provide valuable information about the possibilities and limits of a given approach in humans by helping to better understand the in vivo binding characteristics of an imaging agent. The results of this study suggest that appropriate outcome measures are important in monitoring disease progression and response to therapeutic approaches in human settings. In this study, both tracers for VOI-based ratio analysis could discriminate the AD transgenic and control groups. However, on kinetic parameters from dynamic data, ^18^F-flutemetamol images could not be used as an indicator to distinguish between AD transgenic and control groups. Moreover, the detection of amyloid PET signal in this early aged mouse model used in this study suggests the sensitivity of the PET imaging bio-marker, suggesting the possibility of early detection of amyloid pathology before the manifestation of behavioral abnormalities.

There are several limitations that should be mentioned. First, the distribution patterns between ^18^F-florbetaben and ^18^F-flutemetamol were compared at a single time point. Therefore, the current data are insufficient to judge the superiority between the two tracers based. In a follow-up study, the scope of the analysis should be extended to cover the comparison of serial and chronological accumulation pattern between ^18^F-florbetaben and ^18^F-flutemetamol.

Second, there are some issues regarding methodological perspectives. Herein, for the shape of the merged atlas to match well with the skull CT, the thresholded CT image was manually fused with the magnetic resonance template. However, limitations could exist regarding the method of manual registration. More accurate, automatic algorithm is required in the further study. Additionally, partial volume correction was not conducted when defining the VOI in the blood input area, because the VOI size of the blood input area was larger than the volumetric PET spatial resolution (0.343 mm^3^). Therefore, the effect of the partial-volume correction should be investigated in a further study.

Moreover, in further studies, the different amyloid isoform structures and the range of fibrillarity influencing PET imaging results should be investigated. Amyloid plaques can be sub-classified according to the presence of dystrophic neuritis or reactive astrocytes and the morphological features as either diffuse, fibrillary or dense core types [36, 37]. In the thioflavin S image in our study, the thioflavin S positive plaque areas were predominantly diffuse rather than compact in terms of morphologic characteristic nature. Diffuse plaques are known to occur early in the disease course and to progress towards typical cored plaques [27, 38]. Dense-core plaques are often observed in AD mouse models, at an advanced age [39]. Morphological and biochemical compositional differences of plaques can influence the affinity binding sites for amyloid imaging tracers. Between ^18^F-florbetaben and ^18^F-flutemetamol, which tracer has higher binding affinity to diffuse type plaques? The answer to this question should be investigated in a further study including an in vitro binding assay. In addition, as plaques are amorphous three-dimensional configurations, further three-dimensional analysis of plaque structures with more precise detection stringency should be required.

Moreover, we have not performed Aβ 1-42 staining along with the Aβ 1-40 staining, in the follow-up of immunohistochemistry, because we simply wanted to demonstrate the establishment of AD mouse model and regarded Aβ 1-40 was better choice. Aβ 1-40 presents the most prominent Aβ isoform in the AD brain, while the Aβ 1-42 shows a substantial increase with specific forms of AD [40, 41]. Moreover, extraordinary expressions in AD mice carrying NSE-controlled APPsw presented that Aβ 1-40 was more prominent than Aβ 1-42 in the APPsw mice [42].

## Conclusion

^18^F-florbetaben and ^18^F-flutemetamol images could distinguish between the AD and control group by both visual and SUVR-based analysis. The ^18^F-florbetaben and ^18^F-flutemetamol images showed disparate character in aspects of visual uptake intensity, quantitative parameters, bio-distribution and relations with neuropathological finding. ^18^F-flutemetamol was more actively metabolized than was ^18^F-florbetaben, although ^18^F-florbetaben presented higher visual uptake intensity, SUVR and close correlation with the pathology.

## Additional file

**Additional file 1.** SUVR values of ^18^F-florbetaben and ^18^F-florbetaben images in both AD transgenic and control group. (1) Basic characteristics of AD transgenic and control mouse model (2) SUVR values of ^18^F-florbetaben image in AD transgenic group, (3) SUVR values of ^18^F-florbetaben image in control group, (4) SUVR values of ^18^F-flutemetamol image in AD transgenic group, (5) SUVR values of ^18^F-flutemetamol image in control group.

## Abbreviations

FBB: ^18^F-florbetaben
FMM: ^18^F-flutemetamol
AD: Alzheimer’s disease
Aβ: amyloid plaque
APP: amyloid precursor protein
CSF: cerebrospinal fluid
FDG: fludeoxyglucose
MRI: magnetic resonance image
MCI: mild cognitive impairment
ND: neurodegenerative diseases
NFT: neurofibrillary tangles
PiB: Pittsburgh compound B
PET: positron emission tomography
RFP: red fluorescent protein
GFP: green fluorescent protein
APPswe: Swedish APP mutation
DAPI: diamidino-2-phenylindole
